# Association of Placental Tissue S100A14 Expression with Histologic Chorioamnionitis in Preterm Birth

**DOI:** 10.3390/diagnostics16182919

**Published:** 2026-09-10

**Authors:** Ah-Young Choi, Ha-Yeon Shin, Jue Young Kim, Heesu Chung, Min-A Kim

**Affiliations:** Department of Obstetrics and Gynecology, Gangnam Severance Hospital, Yonsei University College of Medicine, Seoul 06273, Republic of Korea; cay423@yuhs.ac (A.-Y.C.); hayeon37@yuhs.ac (H.-Y.S.); forsythia410@yuhs.ac (J.Y.K.); chungheesu@yuhs.ac (H.C.)

**Keywords:** S100A14, chorioamnionitis, placenta, preterm birth, biomarker

## Abstract

**Background/Objectives:** S100A14 has been implicated in cellular signaling and tissue remodeling, but its relationship with histologic chorioamnionitis (HCA) remains unknown. We evaluated its placental expression and discriminatory performance for HCA in preterm birth. **Methods:** This retrospective cohort included 233 women with singleton spontaneous preterm births. Placental tissues were evaluated by immunohistochemistry for S100A14, MMP-2, ERK, and p38. Expression was compared by HCA status, and correlations were evaluated using Spearman coefficients. Multivariable logistic regression was adjusted for gestational age at delivery and preterm prelabor rupture of membranes (PPROM) status. Discriminatory performance was assessed using receiver operating characteristic analysis. **Results:** S100A14 scores were higher in placentas with HCA than without HCA (5.61 vs. 2.97, *p* < 0.001) and remained independently associated with HCA after adjustment (adjusted odds ratio per 1-point increase, 1.50; 95% confidence interval, 1.31–1.71; *p* < 0.001). MMP-2 expression was also elevated in HCA, whereas ERK and p38 expression did not differ by HCA status. S100A14 correlated most strongly with MMP-2 (ρ = 0.531, *p* < 0.001). Among 173 participants with complete data, the areas under the curve were 0.780 for placental S100A14, 0.717 for placental MMP-2, and 0.660 for maternal serum CRP at admission; the difference between S100A14 and CRP was statistically significant (*p* = 0.023), whereas the difference between S100A14 and MMP-2 was not (*p* = 0.126). At an optimal cutoff of 5.0, sensitivity was 55.2% and specificity was 80.2%. **Conclusions:** Placental S100A14 expression was independently associated with HCA and showed moderate tissue-level discriminatory ability for HCA. Its modest sensitivity limits its utility as a stand-alone diagnostic marker. Further studies are required to determine whether S100A14 or related molecular signals can be detected and validated in specimens obtainable before delivery.

## 1. Introduction

Chorioamnionitis is defined as inflammation of the amniochorionic membranes of the placenta, arising from microbial invasion of the amniotic cavity or from sterile intra-amniotic inflammation in the absence of demonstrable microorganisms [1,2]. Histologic chorioamnionitis (HCA) is found in 40–70% of preterm births complicated by preterm prelabor rupture of membranes (PPROM) or preterm labor, and in 1–13% of term births. Chorioamnionitis is a major risk factor for spontaneous preterm birth and for neonatal infection-related morbidity and mortality, including intracranial hemorrhage, periventricular leukomalacia, chronic lung disease, cerebral palsy, and perinatal death [1,3,4].

Because HCA is frequently subclinical, a definitive diagnosis currently relies on histopathologic examination of the placenta after delivery, which cannot inform antenatal management. Amniocentesis for microbial culture or measurement of amniotic fluid leukocyte counts and inflammatory cytokines can provide indirect evidence of intra-amniotic infection, but this approach is invasive and carries a risk of complications such as membrane rupture or iatrogenic preterm delivery, and culture results are not available rapidly enough to guide acute obstetric decision-making. Maternal serum C-reactive protein (CRP) is widely used as an ancillary marker, but its sensitivity and specificity for HCA are limited [5]. These limitations underscore the importance of characterizing molecular changes associated with placental inflammation and identifying placental tissue markers related to HCA.

S100A14 belongs to the S100 family of low-molecular-weight (9–14 kDa) EF-hand-type calcium-binding proteins, a family of more than 20 members with diverse intracellular and extracellular regulatory functions [6,7]. Extracellular S100A14 has been reported to interact with the receptor for advanced glycation end products (RAGE), whereas separate experimental studies have linked S100A14 to the regulation of MMP-2 expression and activity through p53-dependent transcriptional mechanisms [8,9]. These observations suggest that S100A14 may participate in inflammatory and tissue-remodeling pathways, although the relevant signaling mechanism in placental tissue remains unknown. Dysregulated S100A14 expression has been reported in several malignant tumors, but its expression and function in placental tissue and chorioamnionitis have not previously been described.

The objectives of this study were therefore (1) to characterize placental S100A14 expression and its association with HCA, including its relationships with MMP-2, ERK, and p38 expression, and (2) to evaluate the tissue-level discriminatory performance of placental S100A14 for HCA and to compare it with that of placental MMP-2 and maternal serum CRP at admission among women with preterm birth.

## 2. Materials and Methods

### 2.1. Study Population

We conducted a retrospective cohort study of 233 women with singleton live spontaneous preterm births between 24 + 0 and 36 + 6 weeks of gestation at Gangnam Severance Hospital, Yonsei University College of Medicine, between January 2020 and December 2024. Eligible participants delivered following spontaneous preterm labor with intact membranes or preterm prelabor rupture of membranes (PPROM) and had archival placental tissue suitable for immunohistochemical evaluation. We excluded multiple gestations, cervical cerclage during the index pregnancy, major fetal structural anomalies, intrauterine fetal death, preexisting maternal or placental disorders, and preterm births attributable to maternal trauma. The study was approved by the Institutional Review Board of Gangnam Severance Hospital (IRB No. 3-2024-0427), which waived the requirement for informed consent because of the retrospective study design.

Maternal and neonatal clinical data were obtained from electronic medical records. Archival formalin-fixed, paraffin-embedded (FFPE) placental specimens collected at delivery were retrieved for immunohistochemical analysis. The original hematoxylin and eosin-stained placental slides were independently re-reviewed by two pathologists, and HCA was diagnosed according to the Amsterdam Placental Workshop Group Consensus criteria [10]. HCA diagnosis and immunohistochemistry (IHC) scoring were performed independently, with each assessment blinded to the results of the other and to clinical data. Staging and grading of the maternal and fetal inflammatory responses and assessment of chorionic vasculitis were not performed. Maternal serum CRP levels were measured, and vaginal swabs were obtained for the detection of Ureaplasma urealyticum and Mycoplasma hominis and the assessment of bacterial vaginosis at admission, before the administration of antibiotics, corticosteroids, or tocolytic agents.

### 2.2. Immunohistochemical Staining and Scoring

Placental tissue was sampled from the paracentral region, approximately 1–2 cm from the umbilical cord insertion, to obtain full-thickness parenchymal sections while avoiding the cord-adjacent central area and peripheral margin. Two to four representative tissue blocks were evaluated per case, using the same sampling protocol across the study cohort. Sections (4.5-μm thick) were cut from FFPE placental tissue blocks and heated at 65 °C for 20 min, followed by deparaffinization in xylene for 15 min. The sections were rehydrated through a graded ethanol series (100%, 90%, and 70%; 5 min each) and washed in deionized water. Heat-induced epitope retrieval was performed in 10 mM citrate buffer (pH 6.0) by microwave treatment for 10 min, and endogenous peroxidase activity was quenched with 3% hydrogen peroxide in methanol for 10 min.

Sections were incubated for 120 min at room temperature with primary antibodies against S100A14 (10489-1-AP, Proteintech, Rosemont, IL, USA; dilution 1:100), MMP-2 (#40994, Cell Signaling Technology, Danvers, MA, USA; dilution 1:200), ERK (#4695, Cell Signaling Technology; dilution 1:500), and p38 (#9212, Cell Signaling Technology; dilution 1:300). Bound antibody was detected using the EnVision™ Rabbit/Mouse system (K5007; Agilent Technologies, Santa Clara, CA, USA) for 60 min, visualized with 3,3′-diaminobenzidine tetrahydrochloride (DAB; K3468; Agilent Technologies), and counterstained with hematoxylin (S3309; Agilent Technologies) for 5 min. Sections processed with the primary antibody omitted served as negative controls. Positive tissue controls were used according to the manufacturers’ recommendations: human breast cancer tissue for S100A14, ERK, and p38, and human cervical carcinoma tissue for MMP-2.

For each marker, staining intensity and percentage positivity were assessed in 5–10 representative high-power fields per slide. For each compartment, a semiquantitative intensity score (0, absent; 1, weak; 2, moderate; 3, strong) and a percentage-positivity score (0, 0–5%; 1, 6–25%; 2, 26–50%; 3, 51–75%; 4, 76–100%) were assigned and multiplied to yield a compartment score. For each placental compartment, the scores assigned by the two pathologists were averaged. The final IHC score for each marker was then calculated as the mean of the averaged amnion and chorion scores. Interobserver agreement for the IHC scoring method was assessed using a two-way random-effects, average-measures intraclass correlation coefficient (ICC [2,k]) across all four markers (S100A14, MMP-2, ERK, and p38) in the full cohort (*n* = 233). Representative staining for each intensity and percentage grade is shown in Supplementary Figure S1.

### 2.3. Statistical Analysis

Continuous variables were compared between women with and without HCA using the Welch independent-samples *t* test, and categorical variables were compared using the chi-square test or Fisher’s exact test, as appropriate. Welch *t* tests were also used to compare S100A14, MMP-2, ERK, and p38 IHC scores across subgroups defined by 9 clinical characteristics. These subgroup analyses were exploratory, and no adjustment was made for multiple comparisons. Because the IHC scores were semiquantitative and bounded, Mann–Whitney U tests were additionally performed as nonparametric sensitivity analyses to assess the robustness of comparisons based on Welch’s *t* tests. Correlations among S100A14, MMP-2, ERK, and p38 expression were evaluated using Spearman rank correlation coefficients (ρ).

Receiver operating characteristic (ROC) curve analysis was used to assess the ability of S100A14, MMP-2, and maternal serum CRP to discriminate between placentas with and without HCA. Areas under the curve (AUCs) were estimated with 95% confidence intervals, and sensitivity, specificity, positive predictive value (PPV), and negative predictive value (NPV) were calculated at the optimal cutoff for each marker, defined as the value that yielded the highest combined sensitivity and specificity. Pairwise differences between AUCs were evaluated using paired ROC curve comparisons.

To assess potential confounding by gestational age and membrane status, multivariable logistic regression was performed with HCA as the outcome and S100A14 score, gestational age at delivery, and PPROM status as independent variables. Associations were reported as odds ratios (ORs) with 95% confidence intervals. The association between S100A14 and HCA was also examined within strata defined by gestational age at delivery (<32 vs. ≥32 weeks) and membrane status (PPROM vs. no PPROM). All tests were two-sided, and *p* < 0.05 was considered statistically significant. As a sensitivity analysis, the multivariable model was additionally adjusted for admission-to-delivery interval and the neutrophil-to-lymphocyte ratio (NLR), and, among participants with available microbiologic data, for bacterial vaginosis and Ureaplasma and Mycoplasma colonization status (each coded as a binary presence/absence variable). Analyses were performed using IBM SPSS Statistics, version 32.0.0.0 (IBM Corp., Armonk, NY, USA).

## 3. Results

### 3.1. Maternal, Pregnancy, and Neonatal Characteristics

Baseline maternal characteristics, including maternal age, BMI, gravidity, parity, number of prior abortions, and cervical length, did not differ significantly between women with and without HCA. The frequency of PPROM and the admission-to-delivery interval also did not differ between the groups. Women with HCA were admitted at an earlier gestational age (30.5 vs. 32.8 weeks; *p* < 0.001) and delivered earlier (30.8 vs. 33.4 weeks; *p* < 0.001) than women without HCA. Neonates born to women with HCA had a lower birth weight (1724 vs. 2072 g; *p* < 0.001), lower 1- and 5-min Apgar scores (4.9 vs. 5.6; *p* = 0.005 and 6.4 vs. 7.2; *p* = 0.003, respectively), and a higher rate of neonatal intensive care unit admission (71.1% vs. 49.6%; *p* = 0.001) (Table 1).

### 3.2. Expression of S100A14 in Relation to Clinical Characteristics

**Figure 1:**
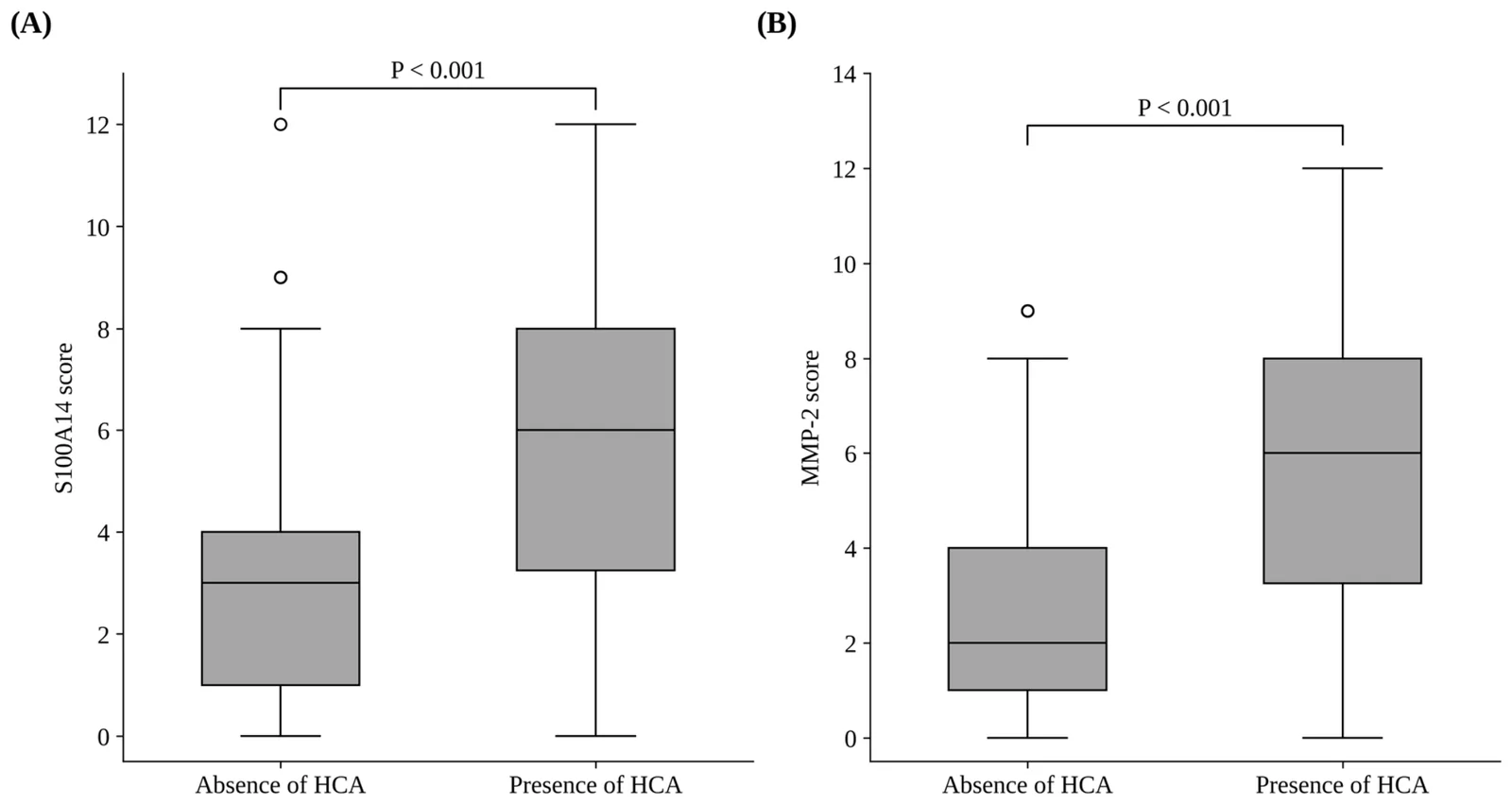
Placental immunohistochemical scores according to histologic chorioamnionitis status: (**A**) S100A14; (**B**) MMP-2. HCA, histologic chorioamnionitis. Boxes represent interquartile ranges, horizontal lines within boxes indicate medians, whiskers indicate values within 1.5 times the interquartile range, and circles indicate outliers.

Interobserver agreement for the IHC scoring method was excellent (ICC [2,k] = 0.84; 95% CI, 0.76–0.90; *p* < 0.001). The mean S100A14 IHC score was significantly higher in placentas with HCA than in those without HCA (5.61 [95% CI, 5.07–6.14] vs. 2.97 [95% CI, 2.53–3.41], *p* < 0.001) (Figure 1A); this finding was unchanged in a nonparametric sensitivity analysis (Mann–Whitney U *p* < 0.001). In exploratory analyses of subgroups defined by 9 additional clinical characteristics, S100A14 expression was higher in placentas from women who delivered before 32 weeks of gestation than in those who delivered at or after 32 weeks (4.83 vs. 3.96, *p* = 0.043; Mann–Whitney U *p* = 0.068) and in those with an admission-to-delivery interval of less than 2 days than in those with an interval of 2 days or longer (4.58 vs. 3.45, *p* = 0.005). S100A14 expression did not differ significantly according to maternal age, BMI, parity, PPROM status, Ureaplasma or Mycoplasma detection status, or bacterial vaginosis (Table 2). Among participants with available microbiologic data (*n* = 179), Ureaplasma detection occurred in 65.6% of women with HCA and 57.3% of those without HCA (*p* = 0.257), Mycoplasma detection in 7.8% and 4.5%, respectively (*p* = 0.360), and bacterial vaginosis in 78.9% and 76.4%, respectively (*p* = 0.690); none of these differences were statistically significant.

### 3.3. Expression of MMP-2, ERK, and p38

The mean MMP-2 IHC score was significantly higher in placentas with HCA than in those without HCA (5.22 vs. 3.06, *p* < 0.001) (Figure 1B), whereas ERK (*p* = 0.347) and p38 (*p* = 0.329) IHC scores did not differ significantly according to HCA status; these findings were unchanged in nonparametric sensitivity analyses (Mann–Whitney U *p* < 0.001, *p* = 0.388, and *p* = 0.212, respectively). In exploratory analyses of subgroups defined by the same 9 clinical characteristics, MMP-2 expression was higher in placentas from women with bacterial vaginosis than in those without bacterial vaginosis (4.29 vs. 3.10, *p* = 0.030; Mann–Whitney U *p* = 0.142). MMP-2, ERK, and p38 expression did not differ significantly according to any of the other clinical characteristics examined (Table 3).

### 3.4. Correlation Among S100A14, MMP-2, ERK, and p38 Expression

Placental S100A14 expression was positively correlated with MMP-2 expression (ρ = 0.531, *p* < 0.001), representing the strongest correlation among the markers examined. S100A14 expression was also positively correlated with ERK (ρ = 0.321, *p* = 0.006) and p38 expression (ρ = 0.329, *p* = 0.004). MMP-2 expression was positively correlated with ERK (ρ = 0.434, *p* < 0.001) and p38 expression (ρ = 0.263, *p* = 0.033), and ERK expression was positively correlated with p38 expression (ρ = 0.339, *p* = 0.004) (Table 4).

### 3.5. Discriminatory Performance for Histologic Chorioamnionitis

**Figure 2:**
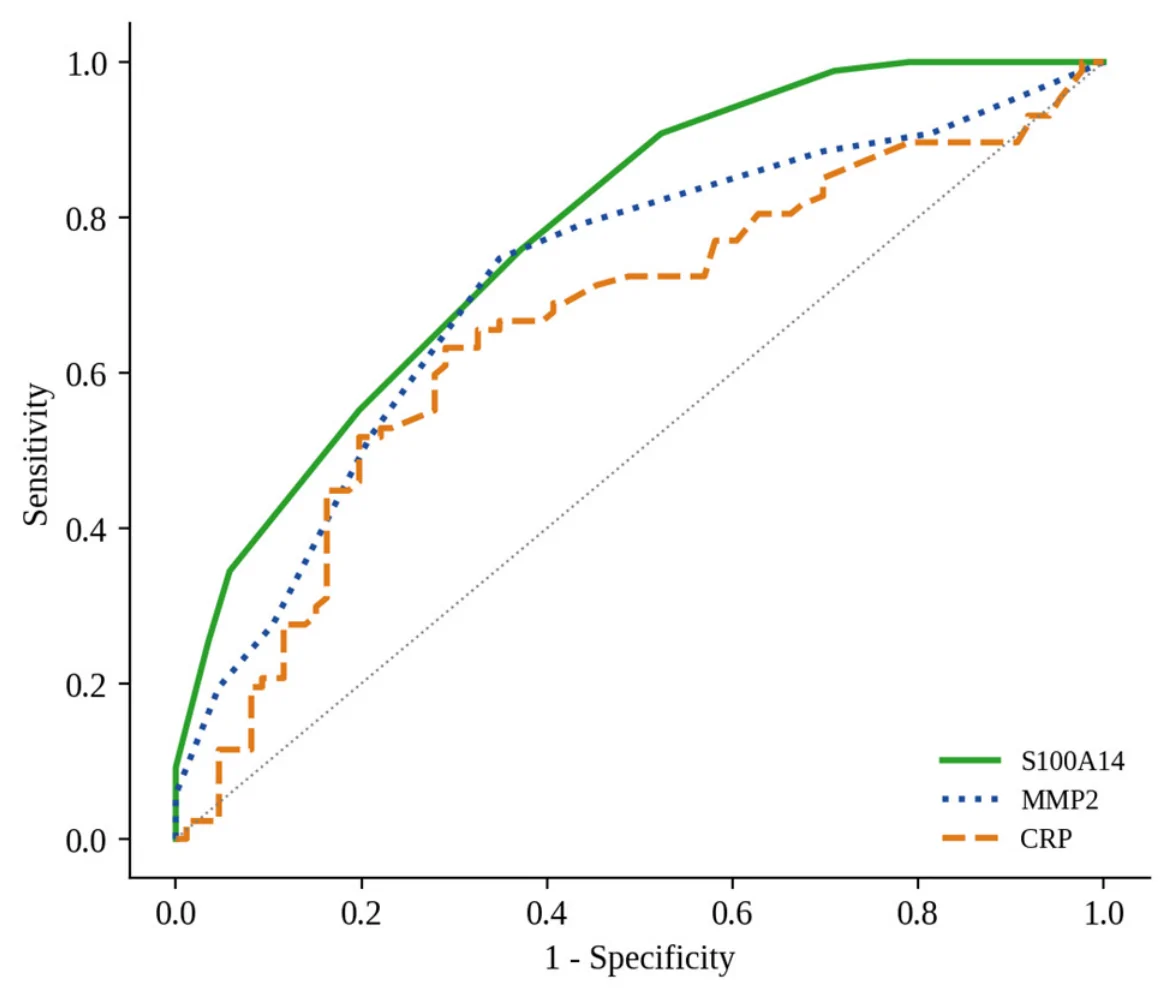
Receiver operating characteristic curves for S100A14, MMP-2, and maternal serum CRP for identifying histologic chorioamnionitis among 173 participants. CRP, C-reactive protein. The diagonal dotted line represents the line of no discrimination (AUC = 0.5).

ROC analysis was performed in 173 participants with complete data for S100A14, MMP-2, and CRP. ERK and p38 were not included because their expression was not significantly associated with HCA status. S100A14 showed the largest AUC (0.780; 95% CI, 0.711–0.849), followed by MMP-2 (0.717; 95% CI, 0.641–0.793) and CRP (0.660; 95% CI, 0.579–0.741) (Figure 2). In paired ROC curve comparisons, the AUC of S100A14 was significantly greater than that of CRP (*p* = 0.023) but did not differ significantly from that of MMP-2 (*p* = 0.126). Although the AUC for placental S100A14 was significantly greater than that for maternal serum CRP at admission, this comparison reflects discriminatory performance within the present cohort and does not indicate clinical superiority, because CRP is antenatally accessible whereas S100A14 was measured in placental tissue obtained at delivery. At an optimal cutoff of 5.0, S100A14 had a sensitivity of 55.2% (95% CI, 44.7–65.2%), specificity of 80.2% (95% CI, 70.6–87.3%), PPV of 73.8% (95% CI, 62.0–83.0%), and NPV of 63.9% (95% CI, 54.5–72.3%) for identifying HCA. At its optimal cutoff of 6.45 mg/L, CRP had a sensitivity of 49.4% and specificity of 80.2% (Table 5). Of the 233 participants, 173 had complete data for S100A14, MMP-2, and CRP and were included in the paired ROC analysis. The remaining 60 were excluded because of missing MMP-2 and/or CRP measurements. Compared with included participants, excluded participants delivered at a later gestational age (231.3 vs. 223.0 days, *p* = 0.019) and had a higher birth weight (2091.5 vs. 1835.8 g, *p* = 0.007), whereas HCA prevalence (45.0% vs. 50.3%, *p* = 0.578) and S100A14 scores (3.68 vs. 4.47, *p* = 0.056) did not differ significantly.

### 3.6. Adjusted and Stratified Analyses by Gestational Age and Membrane Status

In multivariable logistic regression with HCA as the outcome, S100A14 score remained independently associated with HCA after adjustment for gestational age at delivery and PPROM status (adjusted OR per 1-point increase, 1.50; 95% CI, 1.31–1.71; *p* < 0.001). The adjusted effect estimate was similar to the unadjusted estimate (OR, 1.47; 95% CI, 1.30–1.66) (Table 6). Consistent associations were also observed in analyses stratified by gestational age at delivery and membrane status: adjusted OR, 1.36 (95% CI, 1.10–1.68) for <32 weeks and 1.56 (95% CI, 1.32–1.85) for ≥32 weeks; and 1.77 (95% CI, 1.42–2.20) for PPROM and 1.29 (95% CI, 1.12–1.50) for no PPROM (all *p* ≤ 0.005). These findings indicate that the association between S100A14 expression and HCA remained robust after accounting for gestational age at delivery and PPROM status. The association remained robust after additional adjustment for admission-to-delivery interval and NLR (adjusted OR, 1.52; 95% CI, 1.33–1.75; *p* < 0.001; *n* = 233). In a sensitivity analysis additionally accounting for bacterial vaginosis and Ureaplasma and Mycoplasma status among participants with available microbiologic data (*n* = 179), the association remained similar (adjusted OR, 1.51; 95% CI, 1.29–1.76; *p* < 0.001). In an exploratory analysis among HCA-positive placentas, S100A14 scores did not differ significantly between cases with and without funisitis (5.08 vs. 5.67, *p* = 0.358; *n* = 13 with funisitis).

## 4. Discussion

To our knowledge, this is the first study to characterize S100A14 expression in the human placenta. Placental S100A14 expression was significantly elevated in HCA, and the association persisted after adjustment for gestational age at delivery and PPROM status. The association between placental S100A14 expression and HCA remained robust in sensitivity analyses incorporating additional available clinical, inflammatory, and microbiologic variables. The concomitant elevation of MMP-2 and its positive correlation with S100A14 provide a tissue-level rationale for further investigation of the relationship between S100A14 and MMP-2 in placental inflammation.

Several members of the S100 protein family, including S100A8 and S100A12, have been investigated in obstetric research as markers or mediators of intra-amniotic inflammation [11,12,13]. These proteins are predominantly associated with neutrophil and monocyte responses and may reflect leukocyte-mediated inflammation [6,11,13]. In contrast, S100A14 is primarily expressed in epithelial tissues [7,14], raising the possibility that it may reflect a different aspect of the inflammatory response at the maternal–fetal interface. However, the cellular source and functional role of S100A14 in placental tissue remain to be determined.

Recent experimental evidence from nonplacental tissue has shown that S100A14 can induce the expression and secretion of CCL2 and CXCL5 through RAGE–NF-κB signaling, thereby promoting myeloid-cell recruitment [15]. Although demonstrated in a malignant tissue context, this finding raises the possibility that S100A14 may be related to inflammatory signaling relevant to HCA; however, whether a similar mechanism operates in placental tissue remains unknown.

Dysregulated S100A14 expression has been reported in various malignant tumors, and S100A14 has been implicated in the regulation of cell proliferation, survival, and invasion through context-dependent mechanisms [8,9,14,16]. MMP-2 has previously been implicated in extracellular matrix remodeling associated with parturition, PPROM, and intra-amniotic infection, providing biological context for its increased expression in placentas with HCA [17]. In the present study, MMP-2 expression was elevated in HCA, and its correlation with S100A14 was the strongest among the marker pairs examined (ρ = 0.531, *p* < 0.001). Previous experimental studies have independently linked S100A14 to RAGE interaction and to the regulation of MMP-2 expression and activity [8,9,18]. However, these findings do not establish a unified S100A14–RAGE–MMP-2 signaling pathway, and the observed correlation in the present study does not establish a mechanistic relationship in placental tissue. Further mechanistic studies are required to clarify these relationships.

Placental S100A14 staining showed the largest AUC for identifying HCA among the three markers evaluated (AUC, 0.780), followed by MMP-2 (AUC, 0.717) and maternal serum CRP at admission (AUC, 0.660). In paired ROC curve comparisons, the AUC of S100A14 was significantly greater than that of CRP (*p* = 0.023) but did not differ significantly from that of MMP-2 (*p* = 0.126). However, because S100A14 and CRP were assessed in different specimens and at different clinical time points, the higher AUC of placental S100A14 does not establish its clinical superiority over CRP. In particular, maternal serum CRP is readily accessible before delivery, whereas S100A14 was measured in placental tissue obtained only at delivery.

In exploratory subgroup analyses, placental S100A14 expression was higher among women who delivered before 32 weeks of gestation and among those with a shorter admission-to-delivery interval, warranting further investigation of its association with the timing of preterm birth.

This study has several strengths, including a relatively large cohort for a placental IHC study and comparable numbers of HCA-positive and HCA-negative cases. HCA was determined by re-review according to the Amsterdam consensus criteria, and HCA assessment and IHC scoring were conducted independently, with the pathologists blinded to clinical data and to the results of the other assessment. The association between S100A14 and HCA remained consistent after multivariable adjustment and across strata defined by gestational age and membrane status. Moreover, the concurrent evaluation of S100A14 and related markers provided biological context for the findings, while paired ROC analyses enabled direct statistical comparison with MMP-2 and CRP among participants with complete data.

This study has several limitations. First, its retrospective, single-center design and restriction to cases with archival placental tissue suitable for IHC may have introduced selection bias and limited the generalizability of the findings. No a priori sample size or power calculation was performed, as the cohort size was determined by the availability of archival tissue over the study period rather than by a prespecified effect size. Although the association between S100A14 and HCA persisted after adjustment and stratification for gestational age at delivery and PPROM status, residual confounding cannot be excluded, particularly because several potentially relevant variables, including clinical chorioamnionitis, duration of membrane rupture, maternal fever, and antibiotic or corticosteroid exposure, were not available for analysis. Second, the IHC cutoff values were derived and evaluated in the same cohort and may therefore overestimate diagnostic performance. Independent prospective validation is required, particularly given the modest sensitivity of S100A14 for identifying HCA. Third, because S100A14 was measured only in placental tissue obtained at delivery, its antenatal diagnostic utility could not be assessed. Fourth, the observational design and absence of functional experiments precluded confirmation of mechanistic relationships among S100A14, RAGE signaling, and MMP-2 in placental tissue. Fifth, the ROC analysis was restricted to participants with complete data for all three markers. Although HCA prevalence and S100A14 scores did not differ significantly between included and excluded participants, excluded participants delivered later and had higher birth weights; therefore, selection bias related to complete-case analysis cannot be excluded. Sixth, detailed staging and grading of the maternal and fetal inflammatory responses and separate assessment of chorionic vasculitis were not available, limiting further evaluation of S100A14 according to HCA severity. Larger prospective multicenter studies are needed to validate S100A14 as a placental tissue marker of HCA and to determine whether related signals can be detected in specimens obtainable before delivery.

Overall, the present findings support S100A14 as a tissue-level marker associated with HCA rather than as an antenatal or stand-alone clinical diagnostic test. Translation into an antenatal biomarker will require detection of S100A14 or a related molecular signal in specimens obtainable before delivery, such as maternal serum, cervicovaginal fluid, or amniotic fluid, followed by validation of its diagnostic performance in an independent, prospective cohort.

## 5. Conclusions

To our knowledge, this is the first study to characterize placental S100A14 expression in relation to HCA among women with preterm birth. Placental S100A14 expression was significantly elevated in HCA and remained independently associated with HCA after adjustment for gestational age at delivery and PPROM status. S100A14 showed moderate tissue-level discriminatory ability for HCA; however, its modest sensitivity limits its utility as a stand-alone diagnostic marker, and its measurement in placental tissue obtained at delivery precludes any current antenatal application. The difference in AUC between placental S100A14 and maternal serum CRP should not be interpreted as evidence of clinical superiority because the markers were assessed in different specimens and at different clinical time points. Further studies are needed to determine whether S100A14 or related molecular signals can be detected and validated in specimens obtainable before delivery. Dedicated mechanistic studies are also required to investigate the relationship between S100A14, RAGE signaling, and MMP-2 in placental inflammation.

## Figures and Tables

**Table 1:** Maternal, pregnancy, and neonatal characteristics according to histologic chorioamnionitis status.

	Absence of HCA (*n* = 119)	Presence of HCA (*n* = 114)	*p*-Value
**Characteristics of the population**			
Maternal age (years)	32.1 ± 3.5	32.0 ± 3.3	0.927
BMI (kg/m^2^)	24.8 ± 3.4	25.0 ± 3.7	0.740
Gravidity	2.0 ± 1.2	2.1 ± 1.2	0.400
Parity	0.5 ± 0.7	0.6 ± 0.7	0.231
Number of abortions	0.5 ± 0.9	0.5 ± 0.8	0.727
Gestational age at admission (weeks)	32.8 ± 2.8	30.5 ± 5.1	<0.001
PPROM	57 (47.9)	66 (57.9)	0.163
Cervical length (cm)	1.3 ± 1.2	1.0 ± 1.2	0.164
**Pregnancy and neonatal outcomes**			
Gestational age at delivery (weeks)	33.4 ± 2.5	30.8 ± 4.9	<0.001
Admission-to-delivery interval (days)	4.0 ± 8.2	2.5 ± 8.8	0.186
Birth weight (g)	2072 ± 686	1724 ± 695	<0.001
Apgar score 1 min	5.6 ± 1.7	4.9 ± 2.0	0.005
Apgar score 5 min	7.2 ± 1.6	6.4 ± 2.0	0.003
NICU admission	59 (49.6)	81 (71.1)	0.001

Values are given as mean ± standard deviation or *n* (%) unless otherwise specified. HCA, histologic chorioamnionitis; BMI, body mass index; PPROM, preterm prelabor rupture of membranes; NICU, neonatal intensive care unit.

**Table 2:** Placental S100A14 expression according to clinical and microbiologic characteristics.

	No.	Mean Score (95% CI)	*p*-Value
**All study subjects**			
Absence of HCA	119	2.97 (2.53–3.41)	
Presence of HCA	114	5.61 (5.07–6.14)	<0.001
**Maternal age (years)**			
<35	179	4.23 (3.79–4.66)	
≥35	54	4.35 (3.53–5.17)	0.793
**BMI (kg/m^2^)**			
<25	134	4.14 (3.64–4.64)	
≥25	97	4.45 (3.84–5.07)	0.435
**Parity**			
0	126	4.02 (3.52–4.52)	
≥1	107	4.53 (3.94–5.13)	0.195
**Gestational age at delivery**			
≥32 weeks	153	3.96 (3.52–4.41)	
<32 weeks	80	4.83 (4.11–5.54)	0.043
**PPROM**			
No	110	4.29 (3.71–4.87)	
Yes	123	4.23 (3.71–4.74)	0.872
**Delivery interval (days)**			
<2	166	4.58 (4.12–5.05)	
≥2	67	3.45 (2.80–4.10)	0.005
***Ureaplasma***			
No growth	69	3.91 (3.27–4.56)	
Growth	110	4.55 (3.94–5.15)	0.157
***Mycoplasma***			
No growth	168	4.29 (3.83–4.75)	
Growth	11	4.45 (2.34–6.57)	0.870
**Bacterial vaginosis**			
Negative	40	3.67 (3.02–4.33)	
Positive	139	4.48 (3.94–5.02)	0.060

CI, confidence interval; HCA, histologic chorioamnionitis; BMI, body mass index; PPROM, preterm prelabor rupture of membranes.

**Table 3:** Placental MMP-2, ERK, and p38 expression according to clinical and microbiologic characteristics.

	MMP-2 Mean IHC Score (95% CI)	*p*-Value	ERK Mean IHC Score (95% CI)	*p*-Value	p38 Mean IHC Score (95% CI)	*p*-Value
**All study** **subjects**						
Absence of HCA	3.06 (2.54–3.59)		2.88 (2.12–3.63)		3.77 (2.86–4.68)	
Presence of HCA	5.22 (4.56–5.88)	<0.001	3.42 (2.53–4.31)	0.347	4.39 (3.49–5.29)	0.329
**Maternal age (years)**						
<35	4.06 (3.57–4.54)		2.88 (2.27–3.48)		4.10 (3.37–4.83)	
≥35	4.28 (3.19–5.37)	0.707	4.15 (2.60–5.71)	0.119	3.93 (2.62–5.25)	0.817
**BMI (kg/m^2^)**						
<25	4.00 (3.42–4.58)		2.77 (2.07–3.47)		3.81 (3.03–4.60)	
≥25	4.25 (3.54–4.96)	0.582	3.64 (2.67–4.61)	0.141	4.41 (3.33–5.48)	0.368
**Parity**						
0	4.01 (3.40–4.63)		3.33 (2.51–4.14)		3.93 (3.06–4.80)	
≥1	4.21 (3.56–4.86)	0.665	2.84 (2.03–3.64)	0.390	4.24 (3.29–5.20)	0.623
**Gestational age at delivery**						
≥32 weeks	3.95 (3.41–4.49)		3.32 (2.56–4.08)		3.79 (2.93–4.66)	
<32 weeks	4.39 (3.62–5.17)	0.354	2.88 (2.00–3.76)	0.448	4.36 (3.41–5.31)	0.375
**PPROM**						
No	3.98 (3.38–4.58)		3.35 (2.48–4.22)		4.28 (3.37–5.19)	
Yes	4.24 (3.58–4.91)	0.558	2.85 (2.11–3.59)	0.379	3.83 (2.93–4.74)	0.480
**Delivery interval (days)**						
<2	4.23 (3.70–4.76)		3.07 (2.42–3.72)		3.82 (3.14–4.49)	
≥2	3.75 (2.92–4.58)	0.328	3.29 (2.00–4.58)	0.754	5.14 (3.42–6.87)	0.144
***Ureaplasma***						
No growth	3.48 (2.73–4.24)		2.89 (1.68–4.11)		3.67 (2.10–5.23)	
Growth	4.40 (3.68–5.13)	0.081	2.70 (1.86–3.55)	0.789	4.19 (3.16–5.21)	0.565
***Mycoplasma***						
No growth	4.07 (3.53–4.61)		2.76 (2.05–3.47)		4.04 (3.14–4.95)	
Growth	3.64 (0.85–6.43)	0.742	3.00 (−0.90–6.90)	0.862	3.60 (0.48–6.72)	0.727
**Bacterial** **vaginosis**						
Negative	3.10 (2.21–3.99)		3.00 (0.98–5.02)		3.67 (0.95–6.38)	
Positive	4.29 (3.67–4.92)	0.030	2.72 (2.00–3.44)	0.778	4.07 (3.18–4.97)	0.753

IHC, immunohistochemistry; CI, confidence interval; HCA, histologic chorioamnionitis; BMI, body mass index; PPROM, preterm prelabor rupture of membranes.

**Table 4:** Correlations among placental S100A14, MMP-2, ERK, and p38 expression.

		S100A14	MMP-2	ERK	p38
**S100A14**	**Rho**	1.000	0.531	0.321	0.329
	**P**	–	<0.001	0.006	0.004
**MMP-2**	**Rho**		1.000	0.434	0.263
	**P**		–	<0.001	0.033
**ERK**	**Rho**			1.000	0.339
	**P**			–	0.004
**p38**	**Rho**				1.000
	**P**				–

**Table 5:** Discriminatory performance of S100A14, MMP-2, and CRP for histologic chorioamnionitis (*n* = 173).

	AUC (95% CI)	Sensitivity (95% CI)	Specificity (95% CI)	PPV (95% CI)	NPV (95% CI)	Cutoff
**S100A14**	0.780 (0.711–0.849)	55.2 (44.7–65.2)	80.2 (70.6–87.3)	73.8 (62.0–83.0)	63.9 (54.5–72.3)	5.00
**MMP-2**	0.717 (0.641–0.793)	51.7 (41.6–62.2)	79.1 (70.4–87.2)	71.4 (60.4–82.5)	61.8 (52.6–70.8)	5.00
**CRP**	0.660 (0.579–0.741)	49.4 (39.0–59.5)	80.2 (71.4–88.1)	71.7 (60.0–83.0)	61.1 (51.9–69.9)	6.45

AUC, area under the curve; CI, confidence interval; CRP, C-reactive protein; PPV, positive predictive value; NPV, negative predictive value. The CRP cutoff is expressed in mg/L; S100A14 and MMP-2 cutoffs are IHC scores. The paired ROC analysis was restricted to 173 participants with complete data for S100A14, MMP-2, and CRP, whereas the multivariable analysis (Table 6) included the full cohort (*n* = 233).

**Table 6:** Multivariable logistic regression for histologic chorioamnionitis.

Variable	Unadjusted OR (95% CI)	*p*-Value	Adjusted OR (95% CI) ^a^	*p*-Value
S100A14 score (per 1-point increase)	1.47 (1.30–1.66)	<0.001	1.50 (1.31–1.71)	<0.001
Gestational age at delivery (per week)	0.78 (0.70–0.86)	<0.001	0.77 (0.69–0.86)	<0.001
PPROM (yes vs. no)	1.50 (0.89–2.51)	0.127	1.59 (0.85–2.95)	0.146

^a^ Adjusted for the other two variables in the model (*n* = 233). OR, odds ratio; CI, confidence interval; PPROM, preterm prelabor rupture of membranes.

## Data Availability

The original contributions presented in this study are included in the article. Further inquiries can be directed to the corresponding author.

## Supplementary Materials

The following supporting information can be downloaded at: https://www.mdpi.com/article/10.3390/diagnostics16182919/s1, Figure S1: Representative immunohistochemical staining used for intensity and percentage-positivity grading.

## Abbreviations

The following abbreviations are used in this manuscript:

AUC	area under the receiver operating characteristic curve
BMI	body mass index
CI	confidence interval
CRP	C-reactive protein
FFPE	formalin-fixed, paraffin-embedded
HCA	histologic chorioamnionitis
IHC	immunohistochemistry
NICU	neonatal intensive care unit
NPV	negative predictive value
OR	odds ratio
PPROM	preterm prelabor rupture of membranes
PPV	positive predictive value
ROC	receiver operating characteristic
