# Supplementary material for: Association of Placental Tissue S100A14 Expression with Histologic Chorioamnionitis in Preterm Birth
Source: Diagnostics (Basel). 2026 Sep 10;16(18):2919. doi: 10.3390/diagnostics16182919 (PMC13605728; doi:10.3390/diagnostics16182919)
Supplement: Supplementary file 1 [file diagnostics-16-02919-s001.zip › Supplementary_Figure_S1.pdf]

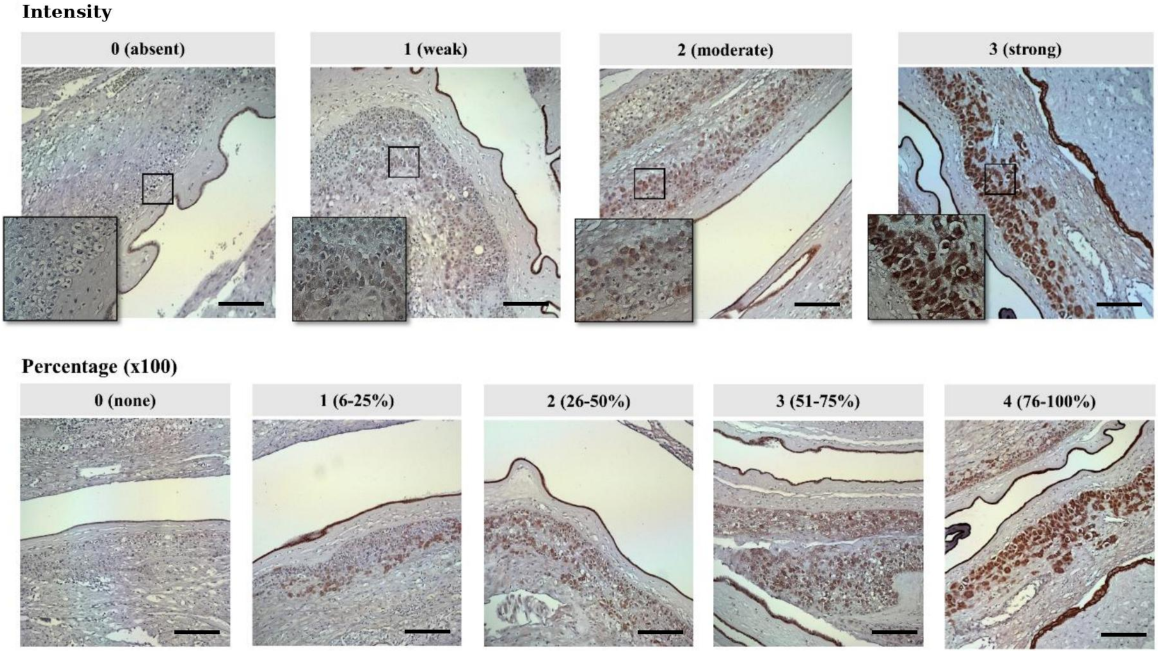

Figure S1. Representative immunohistochemical staining used for intensity and percentage-positivity grading

Representative photomicrographs illustrating the four-point intensity scale (0–3, top row) and five-point percentage-positivity scale (0–4, bottom row) used for immunohistochemical scoring. Boxed regions in the intensity panels are shown at higher magnification (×400). Scale bars = 100 μm in all main panels.
